# Comparison of confound adjustment methods in the construction of gene co-expression networks

**DOI:** 10.1186/s13059-022-02606-0

**Published:** 2022-02-03

**Authors:** Alanna C. Cote, Hannah E. Young, Laura M. Huckins

**Affiliations:** 1 Pamela Sklar Division of Psychiatric Genomics, Icahn School of Medicine at Mount Sinai, New York, NY 10029 USA; 2 Department of Psychiatry, Icahn School of Medicine at Mount Sinai, New York, NY 10029 USA; 3 Department of Genetics and Genomics, Icahn School of Medicine at Mount Sinai, New York, NY 10029 USA; 4 Icahn Institute for Genomics and Multiscale Biology, Icahn School of Medicine at Mount Sinai, New York, NY 10029 USA; 5 Seaver Autism Center for Research and Treatment, Icahn School of Medicine at Mount Sinai, New York, NY 10029 USA; 6 Mental Illness Research, Education and Clinical Centers, James J. Peters Department of Veterans Affairs Medical Center, Bronx, NY 10468 USA

**Keywords:** Co-expression, Confound, Covariate, Batch effects, RNA-seq, Normalization, Module discovery, Complex traits

## Abstract

Adjustment for confounding sources of expression variation is an important preprocessing step in large gene expression studies, but the effect of confound adjustment on co-expression network analysis has not been well-characterized. Here, we demonstrate that the choice of confound adjustment method can have a considerable effect on the architecture of the resulting co-expression network. We compare standard and alternative confound adjustment methods and provide recommendations for their use in the construction of gene co-expression networks from bulk tissue RNA-seq datasets.

**Supplementary Information:**

The online version contains supplementary material available at 10.1186/s13059-022-02606-0.

## Background

Large-scale gene expression studies are often subject to technical and biological sources of expression variation including effects of batch, sample characteristics, and environmental factors. Identifying and correcting for these potential confounders is a crucial step in data preprocessing and can improve researchers’ ability to quantify biological signals of interest [1, 2]. Confounding factors can be documented sources of expression variation (known covariates), or derived empirically from the expression dataset (hidden covariates), and adjusting for these factors has become common practice in many population-level gene expression studies. While the benefits of confounding factor correction have been well-characterized in analyses of differential expression and expression quantitative trait locus mapping [2–5], the effects of confounding factor correction on studies of gene co-expression are less well understood (although see [6, 7]). This is because confounding factors are difficult to distinguish from gene co-expression, as both variables induce patterns of correlation between genes. In fact, in a well-controlled study, hidden factors are likely to represent biological patterns of gene co-expression in the data [8]. Because distinguishing regulatory effects from artifacts is difficult, researchers have historically performed no data correction or known covariate adjustment alone before conducting a co-expression analysis [8–11]. More recently, a series of alternative confound adjustment methods have been proposed, designed to correct the expression dataset for confounding factors while retaining patterns of co-expression [7, 12, 13].

In this study, we evaluate standard and alternative confound adjustment methods in the construction of gene co-expression networks. Using seven diverse tissue datasets from the Genotype-Tissue Expression project (GTEx) and CommonMind Consortium (CMC) [3, 14], we identify co-expression networks after adjustment using six data correction approaches [1, 7, 12, 13]. To aid researchers in future use of these data correction methods, we present the global and local structure of networks derived from each preprocessed dataset, and assess the accuracy of these networks against high-confidence human gene network references [15–17].

## Results and discussion

Our analyses were conducted using GTEx v8 subcutaneous adipose, skeletal muscle, spleen, small intestine-terminal ileum, heart-left ventricle and whole blood tissue datasets, and CommonMind Consortium dorsolateral prefrontal cortex (DLPFC) data, with sample sizes ranging from 174 to 706 individuals. Each dataset underwent preliminary preprocessing including between-sample normalization, gene-level filtering, and gene outlier removal. We applied six data correction procedures to each dataset: (1) no correction, (2) known covariate adjustment, (3) probabilistic estimation of expression residuals (PEER) [1], (4) confounding factor estimation through independent component analysis (CONFETI) [12], (5) removal of unwanted variation (RUVCorr) [7], or (6) principal component adjustment (PC) [13]. RUVCorr, CONFETI, and PC adjustment are three alternative data correction approaches designed to identify and remove hidden confounds while retaining patterns of co-expression in the dataset. We compare these approaches to one popular standard method of hidden confound adjustment (PEER), known covariate adjustment, and a baseline uncorrected dataset (see the “Methods” section). We generated unsigned weighted co-expression networks for each dataset through calculation of the Pearson correlation between gene pairs, defining an edge as an absolute correlation coefficient > 0.5. We also identified co-expression modules using three module detection methods (Additional Files 1, 2, 3): weighted gene correlation network analysis (WGCNA), multiscale embedded gene co-expression network analysis (MEGENA), and independent component analysis (ICA) [18–20].

We assessed the impact of each data correction approach on the architecture of resulting co-expression networks through calculation of fundamental network statistics, including node density, clustering coefficients, and standard module properties [21, 22]. None of these measures alone are sufficient to evaluate the accuracy of each co-expression network following confound adjustment; we report these descriptive statistics to provide an understanding of the size and structure of networks derived from each data correction approach.

**Fig. 1:**
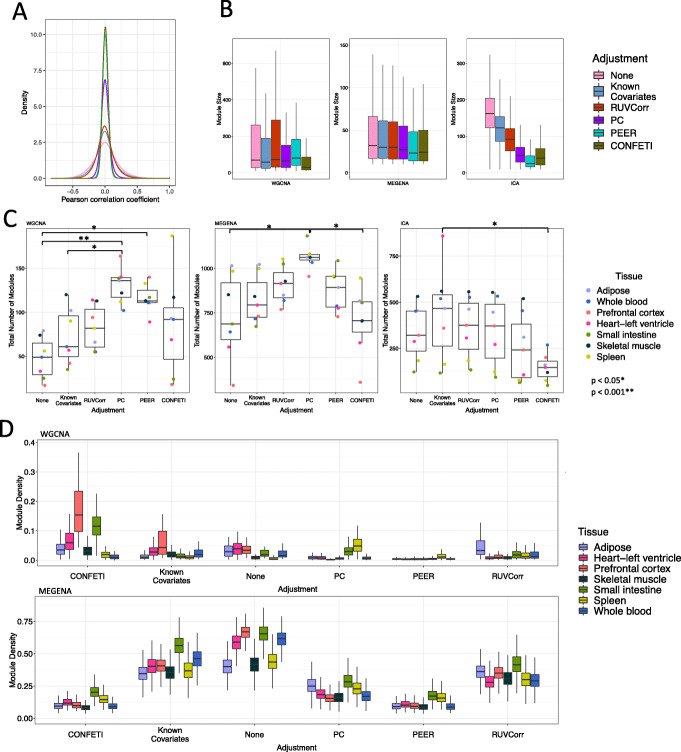
**A** Distribution of gene-gene correlations for 5000 randomly selected genes in the skeletal muscle tissue dataset. We observe significant differences in distribution of gene-gene correlations between adjustment methods across tissues (Pairwise K-S test *D* = 0.018–0.361, all *p* < 0.0001). **B** Box plots showing module size for each module detection method. There is a significant difference in module size between adjustment methods for WGCNA, MEGENA, and ICA-derived modules (all Kruskal-Wallis test *p* < 0.0001). **C** Box plots showing the total number of modules detected. *p*-values are provided in figure for significant pairwise Tukey HSD tests. **D** Box plots showing intramodular density across confound adjustment methods and tissue datasets. Similar to module connectivity, module density measures how tight or cohesive genes are within a group, and is equal to the mean adjacency of a module [22]. Unlike MEGENA and WGCNA, ICA is not a clustering module detection method rooted in the pairwise similarity between genes; therefore, intramodular density was not calculated for ICA-derived modules. Outlier points are omitted for ease of visualization in panels B and D

First, we plotted the distribution of gene-gene correlations for 5000 randomly selected genes from each adjusted dataset. Given that only a small proportion of genes in any random sampling will show functional relationships, we expect the distribution to be normally distributed and centered around zero [7]. The spread of each distribution shows the degree to which patterns of correlated expression have been removed from the dataset. Across tissues, CONFETI and PEER adjustment result in the highest proportion of null gene-gene correlations, while known covariate adjustment, RUVCorr, and no data correction result in the lowest proportion of null gene-gene correlations, indicating that CONFETI and PEER adjustment result in smaller co-expression networks with fewer gene-gene relationships than other adjustment methods (Fig. 1A, Additional File 4: Fig. S2, Additional File 5). Degree distribution and clustering coefficients also differ between adjustment methods, with genes in CONFETI and PEER-adjusted networks showing fewer network neighbors and higher clustering coefficients (Additional File 4: Fig S3-4). We found considerable variation in module size, density, and total module number between tissue type and data correction method (Fig. 1B-D); most notably, modules identified from CONFETI and PEER-adjusted data tend to be smaller and less variable in size (Fig. 1B, Additional File 4: Fig S5). MEGENA modules identified following known covariate adjustment, RUVCorr, or no data correction demonstrate higher intramodular density (i.e., connectedness) across tissues than other correction methods. Additionally, WGCNA modules identified after PEER adjustment tend to be poorly connected, while WGCNA modules identified by CONFETI are particularly densely connected (Fig. 1C). Similarity of modules identified after each data correction method is provided in Additional File 4: Figure S6-8. Overall, there is some overlap among modules identified after known covariate adjustment, RUVCorr, PC, and no data correction (Jaccard index> 0.5) and less overlap between these and modules identified using CONFETI and PEER.

**Fig. 2:**
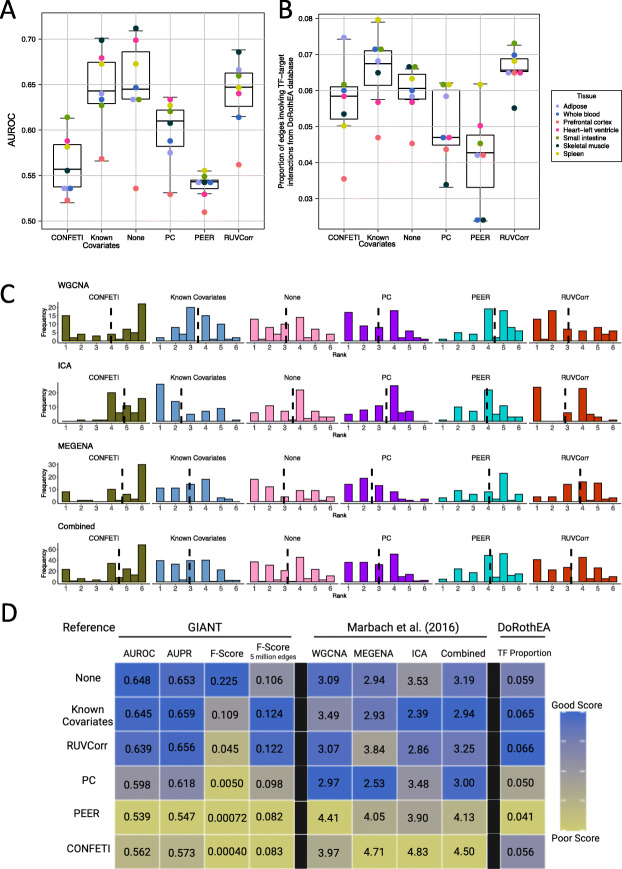
Comparison of covariate adjustment methods. **A** Area under the curve (AUROC) scores for performance evaluation of each adjustment method. **B** Proportion of edges in each global co-expression network that represent TF-target gene interactions from the DoRothEA gene set resource. **C** Aucodds scores for performance evaluation of each adjustment method per module detection method. Each histogram shows the distribution of aucodds score rankings by each cut-off-tissue combination (1 = best-performing method, highest aucodds score), with dashed lines marking the mean rank. **D** Summary of performance evaluation results. Heatmap colors reflect the average score of a performance measure across tissues (scaled from 0 to 1). Module comparison to Marbach et al. resource [16] shows average aucodds score rankings (**C**). The average scores for each evaluation measure are provided in their respective cells

Next, we evaluated the sensitivity and specificity of each correction method through comparison to two high confidence tissue-specific gene network references. First, following Somekh et al. [6], we compared gene-gene co-expression to true positive and negative gene pairs obtained from an external network resource (the Genome-Scale Integrated Analysis of Networks in Tissues (GIANT) [15]). For each expression dataset we (1) selected high probability true positive and true negative GIANT gene pairs, (2) identified coefficients and FDR-adjusted Pearson correlation *p*-values for the corresponding gene pairs in GTEx or CMC, and (3) compared adjusted *p*-values against GIANT network gene pairs to generate receiver operating characteristic curves and calculate the area under the curve (AUROC). RUVCorr, known covariate adjustment, and no data correction perform similarly on this evaluation metric, while PC, PEER, and CONFETI adjustment result in lower AUROC scores than unadjusted data (Fig. 2A). These results are similar when we apply alternative measures of classification performance (Additional File 4: Fig. S9) and alternative cut-offs for co-expression network construction (Additional File 4: Fig. S10).

We also performed comparative network analysis at the modular level, through comparison of co-expression modules in this study and tissue-specific transcriptional regulatory circuits derived from transcription factor motifs, promoter, and enhancer activity information from the FANTOM5 consortium [16, 23, 24]. Co-expression modules were tested for enrichment in gene groups under regulation by the same transcription factor. We summarized the performance of each dataset using an “aucodds” score [25]. This score represents both the number of target gene groups with a significant enrichment result, and the extent to which targets of each regulator are enriched in a given module. Despite substantial variation in performance across tissues, adjustment using RUVCorr, PC, known covariates, and no data correction performed similarly on this evaluation metric, while CONFETI and PEER-adjusted data resulted in poorer overall performance as measured by the average aucodds score ranking (Fig. 2C, Additional File 4: Fig, S11-12).

Because CONFETI was designed to retain only patterns of co-expression associated with common genetic variation, we considered that CONFETI is effectively capturing only genetically regulated co-expression modules in our analysis, leading to poor overall representation of the reference gene networks. To investigate this, we calculated the proportion of modules per dataset that showed significant enrichment for (1) shared TF-targets from Marbach et al. [16] and (2) gene sets from Gene Ontology [26], Reactome [27], and KEGG [28] pathway databases (Additional File 4: Fig. S13). Overall, modules identified from CONFETI-adjusted data are less represented in the Marbach et al. reference network and major pathway databases than other correction methods.

Finally, we compared global co-expression networks to DoRothEA, a curated gene set of TF-target gene relationships derived from literature review, ChIP-seq data, TF binding site motifs, and gene expression [17]. Because DoRothEA is tissue-agnostic, and gene-gene relationships from this resource likely have specific cellular and environmental contexts, we cannot apply performance evaluation measures like AUROC or AUPR that consider both expected true and false gene-gene relationships. Instead, we measured the proportion of gene-gene relationships in each network involving TF-target gene interactions from this database (Fig. 2B). Consistent with results in Fig. 1A, known covariate-adjusted, RUVCorr-adjusted, and baseline unadjusted datasets result in co-expression networks with the highest proportion of edges with supporting evidence from the DoRothEA resource. This result is largely robust to choice of cut-off for a co-expression network edge (Additional File 4: Fig. S14).

A summary of performance evaluation results across tissues is provided in Fig. 2D. This study presents multiple lines of evidence suggesting that CONFETI and PEER adjustment may not be appropriate before co-expression network analysis; both result in particularly sparse networks with weaker representation of high-confidence reference gene-gene relationships compared to other adjustment methods. This poor performance of PEER adjustment is consistent with Somekh et al. [6]. PEER adjustment was designed and tested principally to improve sensitivity in differential expression and *cis*-eQTL studies, not to optimize co-expression analysis. It is likely that some PEER factors represent patterns of biological co-expression; therefore, adjusting for these may reduce researchers’ ability to derive informative co-expression networks. In fact, prior studies have interpreted PEER factors as gene co-expression and used PEER to study genotype effects on gene co-regulation [29, 30].

As mentioned previously, CONFETI was designed to retain patterns of co-expression associated with genotype (i.e., broad impact eQTL). In the original proposal of this method, CONFETI was effective for simulated datasets in removing confounding factors while retaining broad impact eQTL, but showed limited success in real datasets, identifying a few replicating broad impact eQTL. We note this approach was designed only to account for non-genetic confounding sources of expression variation. In practice, some confounds that may be of interest to researchers can also show association with genetic variation, such as cell type proportions [31].

In addition, networks constructed from PC-adjusted datasets show intermediate performance on two of three evaluation measures, providing evidence that PC adjustment may overcorrect the expression dataset. These results contradict Parsana et al., which suggests improved performance of PC-corrected datasets compared to known covariate-corrected and baseline unadjusted data. To further explore this, we evaluated the performance of each adjustment method through calculation of the false discovery rate of WGCNA co-expression modules, as in Parsana et al. Despite using different ground truth references, we also observe a reduced FDR for PC-corrected data for two out of three shared GTEx tissues (Additional File 4: Figure S15). Because performance measures in the present study consider the rate of both true positive and negative findings, we also calculated the false negative rate of WGCNA modules and find an inflated FNR for modules derived from PC-corrected data. This result demonstrates that the rate of type 1 error will be low for sparse networks from over-corrected data, highlighting the importance of additional performance metrics that consider both expected true positive and negative network edges. Also, in this study, we adjusted for the number of principal components as suggested by Parsana et al. in the original proposal of this method. We note however that in some previous co-expression network analyses, adjusting for fewer principal components (typically < 10) effectively removes systematic noise from the dataset without overfitting [6, 32, 33], suggesting that the optimal number of principal components to correct for remains an open question.

Lastly, we found that despite differences in network structure, RUVCorr correction, known covariate adjustment, and no data correction all performed similarly in this study. Although we would theoretically expect that correction at least for known technical factors would improve the accuracy of co-expression networks, there is conflicting evidence that this is the case in practice [6, 13], and in the present study, no form of data correction substantially improved the accuracy of co-expression networks as compared to unadjusted data.

## Conclusions

This study suggests that choice of covariate adjustment can have considerable effects on the structure and accuracy of the resulting co-expression network. PEER and CONFETI adjustment may overcorrect the expression dataset, removing patterns of biological co-expression of potential interest, and are not recommended for researchers interested in comprehensive co-expression network identification. Conversely, RUVCorr and known covariate adjustment appear to be suitable methods of preprocessing before co-expression analysis, as these methods correct for unwanted effects on expression with no appreciable loss in co-expression signal.

The data correction and module detection methods used in the present study were tested using gene expression from bulk tissue samples. Further research is needed to understand whether these methods are effective in cell-type specific or single-cell expression datasets, as sources of expression heterogeneity and patterns of co-expression likely differ in these data types [34]. Further work is also needed to understand whether these results extend to additional methods of global and modular network analysis.

## Methods

An illustration of the design of this study is provided in Additional File 4: Fig. S1 1.

### GTEx datasets

We tested performance of each covariate adjustment method using six tissue datasets from the Genotype-Tissue Expression (GTEx) project version 8 release: whole blood, skeletal muscle, spleen, heart-left ventricle, subcutaneous adipose, and small intestine-terminal ileum. Gene read counts and TPMs were downloaded from https://gtexportal.org/home/datasets. The GTEx genome sequencing data were obtained from dbGaP at http://www.ncbi.nlm.nih.gov/gap through accession number phs000424.v8.p2. Standard RNAseq preprocessing steps were applied to each tissue dataset as follows: (1) TMM normalization of read counts using edgeR and conversion to log2CPM values, (2) gene-level filtering based on threshold of > 0.1 TPM in ≥ 20% of samples and ≥ 6 unnormalized reads in ≥20% of samples, (3) winsorization of expression values, setting values in specific samples that deviate > 3 standard deviations from other samples to 3 standard deviation limit. For application of the CONFETI method, GTEx whole genome sequencing data pruned at R-squared = 0.7 and with MAF ≥ 0.01 was also provided as input.

### CMC dataset

Preprocessing of the dorsolateral prefrontal cortex CMC gene expression dataset was performed largely in accordance with Fromer et al. [8]. This included filtering of lowly expressed genes (> 1 CPM in at least 50% of samples), conditional quantile normalization, and winsorization of gene counts, setting values in specific samples that deviate > 3 standard deviations from other samples to 3 standard deviation limit. Sample outliers were removed based on (1) visual inspection of the first two principal components of the full gene expression matrix and (2) interarray correlation, removing samples with correlation less than 3 standard deviations below mean for the dataset. For application of the CONFETI method, CMC dosage data imputed to the TOPMed reference panel, pruned at R-squared = 0.7 and with MAF ≥ 0.01 was also provided as input.

### Covariate adjustment

The following covariate adjustment methods were tested:

1. None: The dataset was not corrected for any known or hidden confounding factor.
2. Known covariates:

GTEx: Information concerning technical factors and sample attributes were downloaded from https://gtexportal.org/home/datasets. Covariates with missing information or zero variance were excluded. We calculated the canonical correlation between remaining covariates; for highly collinear variables (coefficient > 0.9 for collinear factors), we selected and retained one variable at random. Next, we calculated the variance in expression attributable to remaining continuous technical factors using the *variancePartition* package. Lastly, each expression dataset was adjusted for continuous technical factors that explained ≥ 1% variation in ≥ 10% of genes, as well as genotype-derived PC1-5, sex, and binned age. A description of covariates adjusted for in each tissue is provided in Additional File 4: Table S2.

CMC: The dataset was adjusted for technical factors as in Fromer et al. [8]. This included adjustment for diagnosis, institution, sex, age of death, postmortem interval, RNA integrity number (RIN), RIN^2^, genotype-derived PC1-5, and a clustered library batch variable.

3) Principal components: It has been proposed that for scale-free networks, i.e., networks where the degree distribution follows a power law, patterns of co-expression are sufficiently sparse that principal components of the expression matrix represent an effective form of confound correction [13]. As suggested by Parsana et al., the number of principal components to consider was determined through a permutation-based approach implemented using the “num.sv” function in the *sva* package [13]. The number of principal components included in adjustment of each tissue is provided in Additional File 4: Table S1. Significant principal components were regressed on each gene using a linear model and expression residuals obtained.
4) PEER: Probabilistic estimation of expression residuals (PEER) is a popular confound correction method that uses a variant on the traditional factor analysis method to infer hidden factors from the gene expression dataset [1, 4]. PEER factors were obtained using default settings through the *peer* package. For each dataset, we adjusted for the number of PEER factors selected to optimize cis-eGene discovery in the latest quantitative trait locus study by the GTEx Consortium [3]: 15 factors for tissues with < 150 samples, 30 factors for tissues with 150–249 samples, 45 factors for tissues with 250–349 samples, and 60 factors for tissues with ≥ 350 samples.
5) CONFETI: Confounding Factor Estimation Through Independent component analysis (CONFETI) is designed to adjust for non-genetic confounding factors while retaining genetically regulated co-expression (i.e., broad impact eQTL) in the expression dataset [12]. Briefly, factors are derived from the full gene expression dataset using independent component analysis. Each independent component is tested for association with genotype in a preliminary broad impact eQTL analysis. Independent components not associated with genotype are considered non-genetic confounding factors and are used in construction of a random effects sample covariance matrix.

We identified genetic and non-genetic independent components using the *confeti* package with default settings. Unadjusted gene expression data for each tissue and genotype pruned at R-squared = 0.7 were provided as input. The number of independent components used as confounding factors for each tissue is provided in Additional File 4: Table S1. Five ancestry PCs were regressed on the expression of each gene in a linear mixed model using the *lrgpr* package with non-genetic confounding factors provided as a sample covariance matrix, and gene expression residuals obtained.

6) RUVCorr: The removal of unwanted variation (RUV) method is a multivariate linear model that estimates systematic noise through factor analysis on an expression matrix of empirically derived negative control genes, i.e., genes in the data with low expression variation that are not expected to be associated with the biological signal of interest (co-expression) [7]. In an attempt to mitigate bias in the case where systematic noise and biological signal of interest are correlated, the RUV method uses ridge regression to estimate the effect of systematic noise on expression and regresses this systematic noise from the expression dataset. The dimensionality of the noise (k) is chosen by the researcher through visual inspection of plots of the distribution of negative and positive control genes in each dataset. A subset of 2000 genes were used as empirically derived negative controls while sodium channel genes, major histocompatibility complex genes, and genes that encode for the protein component of the ribosome were used as positive controls (Additional File 4: Fig. S16-22, positive control gene groups provided in Additional File 6). The ridge parameter (υ) is chosen through visual inspection of relative log expression plots (Additional File 4: Fig. S23-24). Optimal parameters will reduce the correlation between random genes, retain correlation between positive control genes, and best retain gene expression variances in the dataset. RUV correction was applied to each dataset using the *RUVcorr* package, and expression residuals obtained. Choice of RUV parameters for each tissue is provided in Additional File 4: Table S1.

### Co-expression module detection

The following module detection methods were used:

1. WGCNA: Each dataset was transformed to a soft-thresholding power β to approximate scale-free topology (choice of power parameter provided in Additional File 7), followed by construction of an unsigned network with a minimum module size of 10 genes. Modules were merged if correlation of their module eigengenes exceeded a Pearson correlation coefficient of 0.75.
2. MEGENA: MEGENA module detection was performed using all default settings.
3. ICA: The *R* fastICA algorithm was applied to each expression dataset using the *logcosh* function for neg-entropy approximation [20]. The number of independent components extracted equaled the number of components that estimated 95% of the variance as calculated by PCA. Inclusion of a gene in an ICA module was determined through false discovery rate (FDR) calculation of source signal weights as in Rotival et al. [35]. FDR estimation was performed using the *R* fdrtool package [36], and genes with an adjusted *p*-value < 0.0001 were added to a module. Modules with fewer than 10 genes were excluded from this study, to be consistent with the minimum module size of 10 genes for WGCNA and MEGENA.

### Gene set enrichment

We tested modules derived from CONFETI-adjusted data for enrichment in gene sets from the Gene Ontology, KEGG, and Reactome databases using the gprofiler2 *R* package v0.2.0. Over-representation of gene sets in each co-expression module was tested using the hypergeometric test, with a custom background defined as the number of genes expressed in each tissue dataset.

### Comparative network analysis

We used the following methods to compare our co-expression network results to external references:

1. AUROC measure: First, we compared our co-expression network results to high probability true positive and negative gene pairs from the GIANT interface. To obtain high probability gene pairs, for each tissue-specific GIANT network, we filtered for genes present in each expression dataset, ranked the network by posterior probability, and kept the top 5000 and bottom 5000 gene pairs as true positive and negative gene pairs, respectively. After filtering, the selected true positive and negative gene pairs represent roughly the top 0.004% and bottom 0.004% of interactions for each reference network. Finally, we calculated the Pearson correlation coefficient and FDR-adjusted *p*-values for the corresponding gene pairs in the expression dataset and compared the adjusted *p*-values against GIANT network gene pairs to generate receiver operating characteristic curves and calculate the area under the curve (AUROC).
2. Aucodds measure: Next, we tested our identified co-expression modules for enrichment of targets of a shared transcription factor. Using the Marbach et al. regulatory networks as a reference dataset [16], each gene module was tested for enrichment in target gene groups at various cut-off weights for a true regulator-target gene relationship through a Fisher’s exact test. For each Fisher’s exact test, the background genome size was defined as the number of expressed genes in that particular GTEx expression dataset. For each significant enrichment result (Holm-adjusted *p* < 0.1), we obtained the maximum odds ratio for every regulator across modules. Finally, the performance of each network was summarized through calculation of an “aucodds score” [25]. The aucodds score is the area under the curve formed by the proportion of regulators with an odds ratio greater than a certain cut-off and the log10 odds ratio cut-off within the OR interval of 1–1000.
3. Proportion of edges with evidence in DoRothEA database: We compared global co-expression networks to the DoRothEA resource [17]. The human reference database was downloaded using the *dorothea* R package v.1.4.1 and filtered to exclude TF-target gene pairs inferred only from gene expression. Then, we measured the proportion of edges in each network involving TF-target gene interactions from this database.

## Supplementary Information

**Additional file 1.** WGCNA module assignment for each gene. Each sheet provides module assignments for a particular tissue dataset.

**Additional file 2.** ICA module assignment for each gene. Each sheet provides module assignments for a particular tissue dataset.

**Additional file 3.** MEGENA module assignment for each gene. Each sheet provides module assignments for a particular tissue dataset.

**Additional file 4.** Supplemental Figures S1-S24. Supplemental Table S1-2.

**Additional file 5.** Number of edges and nodes in each global co-expression network.

**Additional file 6.** Positive control gene groups used for evaluation of RUVCorr parameters.

**Additional file 7.** Choice of power parameter for each WGCNA co-expression module search.

**Additional file 8.** Review history.

## Data Availability

Tissue-specific GIANT networks can be downloaded from http://giant.princeton.edu/download/. Marbach et al. (2016) networks constructed from FANTOM5 Consortium data can be downloaded from https://www.synapse.org/#!Synapse:syn4974692. The GTEx genome sequencing data used for the analyses in this manuscript were obtained from dbGaP at http://www.ncbi.nlm.nih.gov/gap through accession number phs000424.v8.p2. All other GTEx datasets supporting this article are available in the GTEx repository, at: https://gtexportal.org/home/datasets. CommonMind Consortium datasets supporting this article are available via the CMC Knowledge Portal: https://www.synapse.org/#!Synapse:syn2759792/wiki/69613.

## Footnotes

1 Created with BioRender.com
