# Supplementary material for: Comparison of confound adjustment methods in the construction of gene co-expression networks
Source: Genome Biol. 2022 Feb 3;23:44. doi: 10.1186/s13059-022-02606-0 (PMC8812044; doi:10.1186/s13059-022-02606-0)
Supplement: Supplementary file 4 — Additional file 4. Supplemental Figures S1-S24. Supplemental Table S1-2. [file 13059_2022_2606_MOESM4_ESM.docx]

**
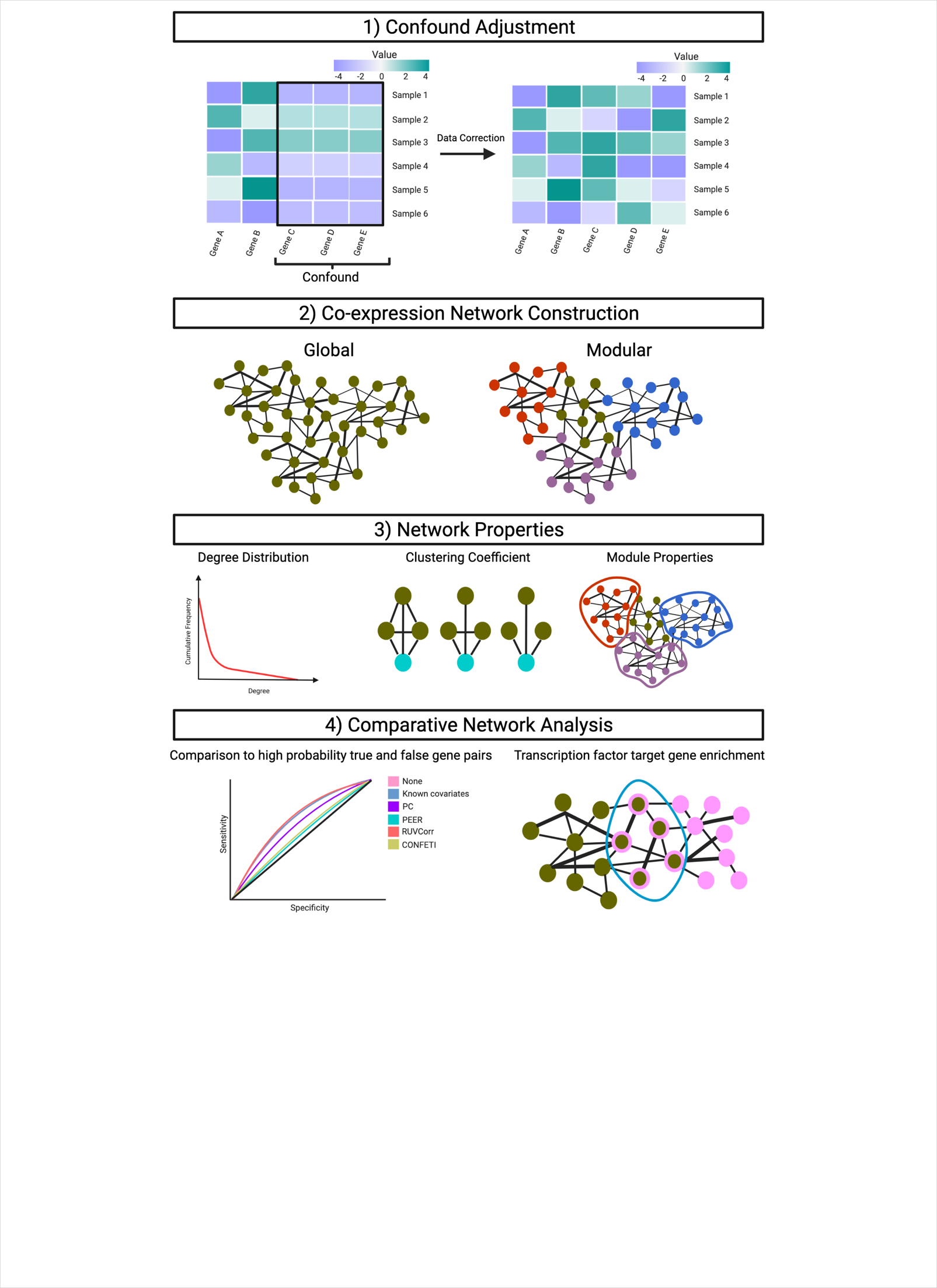
**

**Figure S1. Illustration of confound adjustment evaluation framework.** Seven tissue datasets were used for this study: whole blood, subcutaneous adipose, spleen, heart-left ventricle, skeletal muscle, small intestine-terminal ileum, and prefrontal cortex. Our framework for assessing confound adjustment was as follows: 1) Adjustment of the gene expression dataset using each data correction approach; 2) Construction of global and modular co-expression networks; 3) Investigation of topological network properties; 4) Evaluation of each data correction approach through comparative network analysis.

**Figure S2.** Distribution of gene-gene correlation for 5,000 randomly-selected genes. A) Adipose, B) Whole Blood, C) Skeletal muscle, D) Small intestine, E) Spleen, F) Heart-left ventricle, G) Prefrontal cortex.

**Figure S3**. Degree distribution of the co-expression network for each data correction-tissue combination. Edges defined as an absolute Pearson correlation coefficient > 0.5. A) Heart-left ventricle, B) Whole blood, C) Spleen, D) Adipose, E) Skeletal muscle, F) Small intestine, G) Prefrontal cortex.

**Figure S4**. Distribution of clustering coefficients for each data correction-tissue combination. Edges defined as an absolute Pearson correlation coefficient > 0.5.

**Figure S5**. Distribution of module size for each data correction-tissue combination.

**Figure S6**. Similarity of WGCNA modules between adjustment methods, as measured by the number of module pairs with Jaccard index > 0.5. Axis label = Adjustment method (Total number of modules)

**Figure S7**. Similarity of ICA modules between adjustment methods, as measured by the number of module pairs with Jaccard index > 0.5. Axis label = Adjustment method (Total number of modules)

**Figure S8**. Similarity of MEGENA modules between adjustment methods, as measured by the number of module pairs with Jaccard index > 0.5. Axis label = Adjustment method (Total number of modules)

**Figure S9**. Area-under-the precision recall curve (AUPR) and F-score for performance evaluation of global co-expression networks. Due to the balanced true positive and negative classes selected for each tissue, AUPR score of the null model is 0.5 across tissues. The F-score is the harmonic mean of precision and recall at a given threshold. Networks where both precision and recall equal zero are omitted from F-score plots. B) F-score for global networks with edge defined as an absolute Pearson correlation coefficient > 0.5. This result is robust to choice of threshold for a true gene-gene relationship (Fig S10). C) F-score for global networks, for top five million network edges excluding self-loops (ranked by absolute Pearson correlation coefficient).


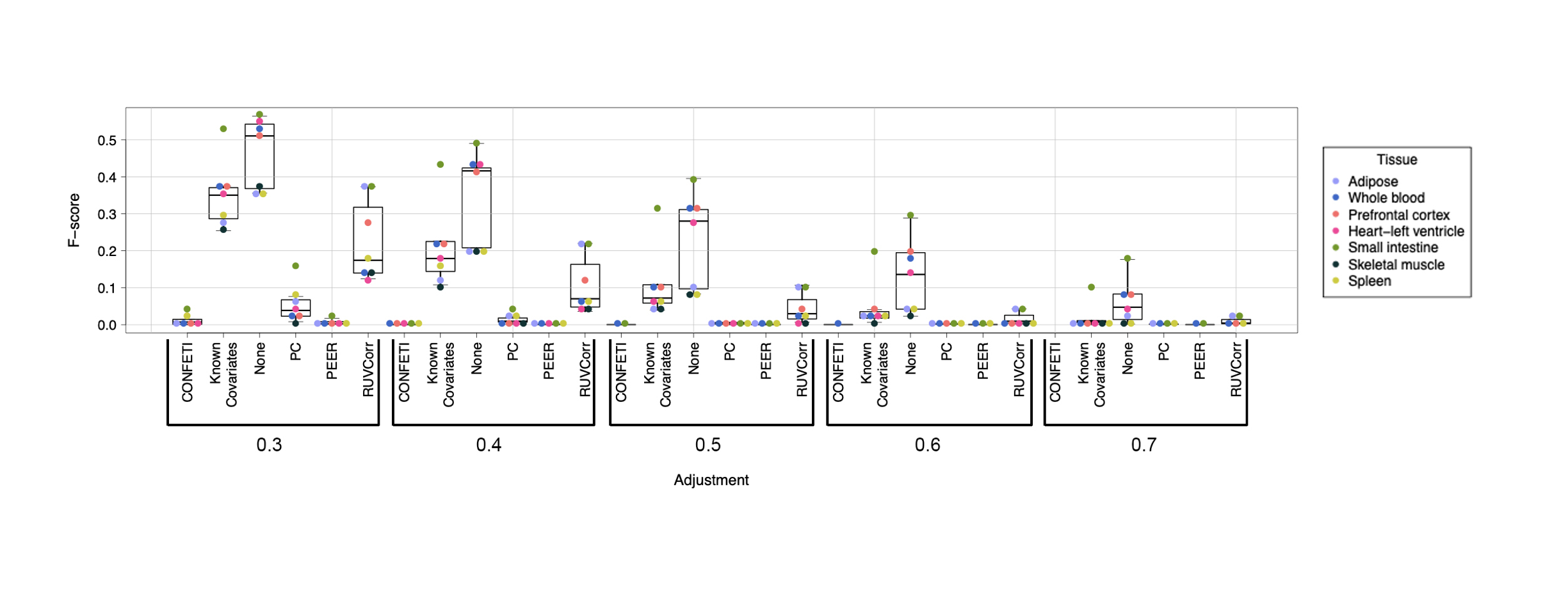
**Figure S10**. F-score for performance evaluation of global co-expression networks. Cut-off for a gene-gene relationship measured by absolute Pearson correlation coefficient (coefficient beneath brackets). Networks where both precision and recall equal zero are omitted from the plot.

**Figure S11**. Aucodds score for performance evaluation of co-expression modules for adipose, heart, and small intestine tissue datasets. Results reported at each cut-off weight for a true transcription factor-target gene relationship.

**Figure S12**. Aucodds score for performance evaluation of co-expression modules for skeletal muscle, spleen, whole blood, and prefrontal cortex tissue datasets. Results reported at each cut-off weight for a true transcription factor-target gene relationship.

**Figure S13**. Top boxplot shows the proportion of WGCNA modules with significant enrichment (FDR 5%) for the targets of at least one regulator, averaged across cut-offs for a true TF-target gene relationship. Bottom boxplots show the proportion of WGCNA modules with significant enrichment (Bonferroni correction for number of modules and gene sets) for at least one gene set in the Gene Ontology, KEGG, and Reactome pathway databases.


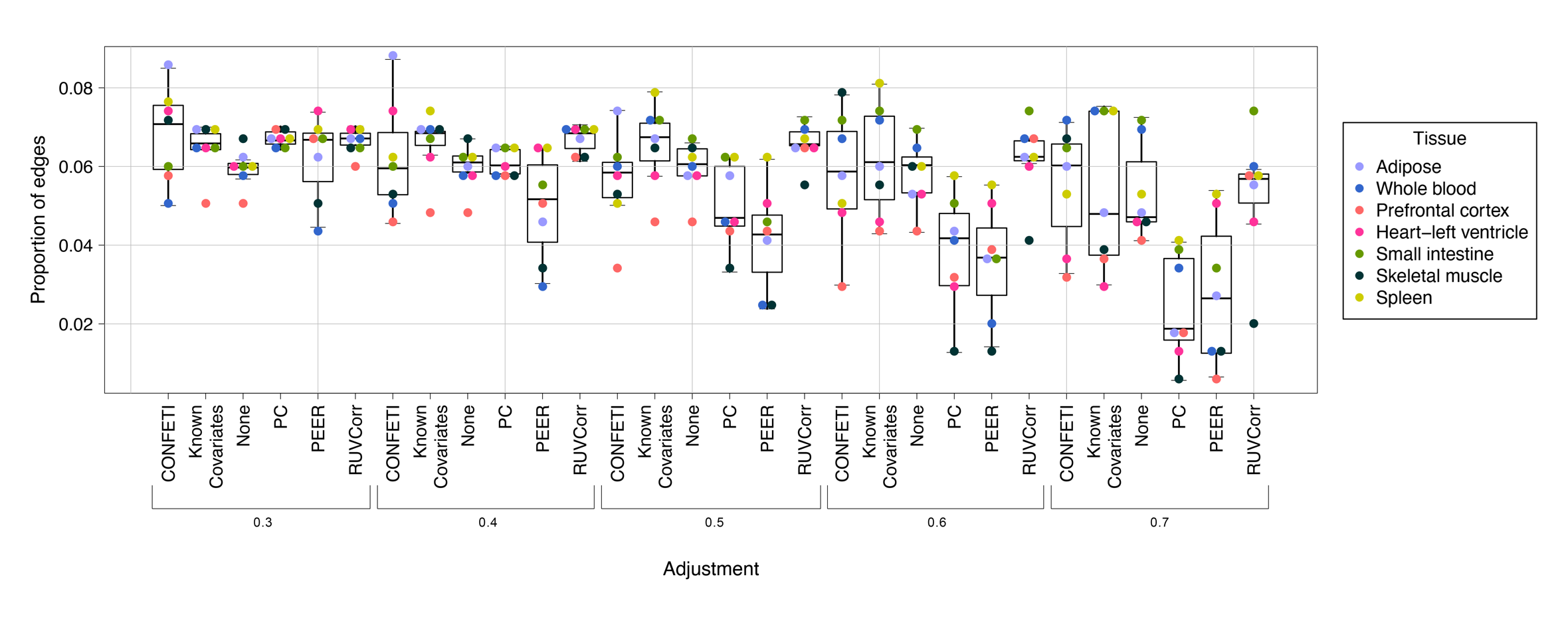
**Figure S14**. Proportion of global network edges involving TF-target interactions from the DoRothEA database. Results are robust to choice of cut-off for a gene-gene relationship, as measured by absolute Pearson correlation coefficient (coefficient beneath brackets).

**Figure S15.** False discovery rate and false negative rate of WGCNA co-expression modules when compared to positive and negative gene pairs from the GIANT reference networks. A gene pair present in the same module and as a positive gene pair in the reference network was considered a true positive, while a gene pair not present in the same module and present as a true negative gene pair in the reference network was considered a true negative.

**Figure S16**. Distribution of gene-gene correlations at different RUVCorr parameter settings. Tissue = skeletal muscle. The underlying histogram shows the distribution of gene-gene correlations for each gene set, calculated using unadjusted data. A) Random genes, B) Sodium channel genes, C) Ribosome genes, D) Major histocompatibility complex (MHC) genes

**Figure S17**. Distribution of gene-gene correlations at different RUVCorr parameter settings. Tissue = adipose. The background histogram shows the distribution of gene-gene correlations for each gene set, calculated using the unadjusted data. A) Random genes, B) Sodium channel genes, C) Ribosome genes, D) Major histocompatibility complex (MHC) genes

**Figure S18**. Distribution of gene-gene correlations at different RUVCorr parameter settings. Tissue = whole blood. The background histogram shows the distribution of gene-gene correlations for each gene set, calculated using the unadjusted data. A) Random genes, B) Sodium channel genes, C) Ribosome genes, D) Major histocompatibility complex (MHC) genes

**Figure S19**. Distribution of gene-gene correlations at different RUVCorr parameter settings. Tissue = heart-left ventricle. The background histogram shows the distribution of gene-gene correlations for each gene set, calculated using the unadjusted data. A) Random genes, B) Sodium channel genes, C) Ribosome genes, D) Major histocompatibility complex (MHC) genes

**Figure S20**. Distribution of gene-gene correlations at different RUVCorr parameter settings. Tissue = spleen. The background histogram shows the distribution of gene-gene correlations for each gene set, calculated using the unadjusted data. A) Random genes, B) Sodium channel genes, C) Ribosome genes, D) Major histocompatibility complex (MHC) genes

**Figure S21**. Distribution of gene-gene correlations at different RUVCorr parameter settings. Tissue = small intestine. The background histogram shows the distribution of gene-gene correlations for each gene set, calculated using the unadjusted data. A) Random genes, B) Sodium channel genes, C) Ribosome genes, D) Major histocompatibility complex (MHC) genes

**Figure S22**. Distribution of gene-gene correlations at different RUVCorr parameter settings. Tissue = prefrontal cortex. The background histogram shows the distribution of gene-gene correlations for each gene set, calculated using the unadjusted data. A) Random genes, B) Sodium channel genes, C) Ribosome genes, D) Major histocompatibility complex (MHC) genes

**Figure S23**. Histogram plots of relative log expression at optimal k parameter and various nu parameters. Each figure includes boxplots of the first and third quartile of difference between gene expression and the median for raw and RUV-adjusted data. A) Skeletal muscle (k=2), B) Whole blood (k=4), C) Heart-left ventricle (k=4), D) Small intestine (k=2).

**Figure S24**. Histogram plots of relative log expression at optimal k parameter and various nu parameters. Each figure includes boxplots of the first and third quartile of difference between gene expression and the median for raw and RUV-adjusted data. E) Spleen (k=4), F) Adipose (k=3), G) Prefrontal cortex (k=2).

**Table S1**. **Sample characteristics and data correction parameters,**

| Tissue | Number of Samples | Number of Genes | RUVCorr | | CONFETI | PC |
| --- | --- | --- | --- | --- | --- | --- |
|  |  |  | k | nu | Number of IC's used as confounding factors | Number of PCs included in adjustment |
| Whole Blood | 670 | 20358 | 4 | 1000 | 348 / 454 | 49 |
| Adipose | 581 | 23976 | 3 | 500 | 386 / 457 | 49 |
| Skeletal muscle | 706 | 21084 | 2 | 500 | 453 / 533 | 53 |
| Small intestine | 174 | 26247 | 2 | 1000 | 105 / 126 | 12 |
| Spleen | 227 | 25537 | 4 | 1000 | 161 / 191 | 28 |
| Heart-left ventricle | 386 | 21412 | 4 | 1000 | 246 / 292 | 32 |
| Prefrontal cortex | 491 | 20559 | 2 | 1000 | 280 / 325 | 27 |

**Table S2. Covariates included in known covariate adjustment, per tissue.**

| **Tissue** | **Covariates** |
| --- | --- |
| Whole blood | Sex, age, PC1-5, RNA integrity number (RIN), total number of transcripts with at least 5 exon mapping reads, total ischemic time, chimeric pairs, exonic rate, fragment length stdev, intergenic rate, number of reads aligning to rRNA regions, intronic rate, mapped unique rate of total, alternative alignments, number of split reads |
| Adipose | Sex, age, PC1-5, autolysis score, RIN, total ischemic time, total number of genes with at least 5 exon mapping reads, fragment length stdev, number of all reads aligning to ribosomal RNA regions, failed vendor QC check, intronic rate, mapped unique rate of total, alternative alignments, base mismatch rate, number of End 1 reads sequenced in the sense direction, rRNA rate |
| Skeletal muscle | Sex, age, PC1-5, RIN, total ischemic time, End 2 mapping rate, chimeric pairs, intragenic rate, exonic rate, number of reads aligning to rRNA regions, total number of transcripts with at least 5 exon mapping reads, alternative alignments, mean fragment length, base mismatch rate, rRNA rate |
| Small intestine | Sex, age, PC1-5, autolysis score, RIN, total ischemic time, time spent in PAXgene fixative, chimeric pairs, intragenic rate, total number of genes with at least 5 exon mapping reads, End 1 mismatch rate, number of reads aligning to rRNA regions, failed vendor QC check, intronic rate, mapped unique rate of total, alternative alignments, mean fragment length, base mismatch rate, number of End 1 reads sequenced in sense direction, rRNA rate, End 1 mapping rate, End 2 % sense |
| Heart-left ventricle | Sex, age, PC1-5, RIN, total ischemic time, intragenic rate, exonic rate, failed vendor QC check, total number of transcripts with at least 5 exon mapping reads, alternative alignments, mean fragment length, split reads, base mismatch rate, number of End 1 reads sequenced in the sense direction, End 1 % sense, rRNA rate, End 1 mapping rate, End 2 % sense |
| Spleen | Sex, age, PC1-5, RIN, autolysis score, total ischemic time, time spent in PAXgene fixative, End 2 mapping rate, chimeric pairs, End 1 mismatch rate, intergenic rate, number of all reads aligning to rRNA regions, failed vendor QC check, total number of transcripts with at least 5 exon mapping reads, intronic rate, End 2 antisense, alternative alignments, base mismatch rate, End 1% sense, rRNA rate, End 1 mapping rate, End 2% sense |
| Prefrontal cortex | Diagnosis, institution, sex, age of death, postmortem interval, RIN, RIN^2^ PC1-5, clustered library batch |
