# Supplementary material for: Comparison of confound adjustment methods in the construction of gene co-expression networks
Source: Genome Biol. 2022 Feb 3;23:44. doi: 10.1186/s13059-022-02606-0 (PMC8812044; doi:10.1186/s13059-022-02606-0)
Supplement: Supplementary file 8 — Additional file 8. Review history. [file 13059_2022_2606_MOESM8_ESM.docx]

Review History

**First round of review**

**Reviewer 1**

**Are you able to assess all statistics in the manuscript, including the appropriateness of statistical tests used?** Yes, and I have assessed the statistics in my report.

**Comments to author:**

This manuscript compares the performances of several confound adjustment methods for co-expression analysis through the applications to six bulk tissue RNA-seq datasets. Some recommendations are provided for the use of these confound adjustment methods, which can be helpful for biologists when conducting co-expression analysis. The manuscript is well written. My major concern is that no significance results are provided to support their comparison results. To name a few:

1. In Fig. 1A and Fig. S2, the densities of Pearson correlation coefficients of various methods are compared, but no p-values are provided. The correlation coefficient difference between any two methods could be simply due to randomness, so I suggest that the authors provide pairwise comparison test results to support their findings.

2. In Fig. 1C and Fig. 1D, are the module sizes and module densities significant different between various methods?

3. In Fig. 2B, the aucodds scores are provided, again, are the differences significant?

**Reviewer 2**

**Are you able to assess all statistics in the manuscript, including the appropriateness of statistical tests used?** Yes, and I have assessed the statistics in my report.

**Comments to author:**

In this paper, the authors present an interesting and useful analysis of how one must correct bulk RNA-seq expression datasets to remove known and unknown confounding factors before performing co-expression network analysis. To this end the authors obtained datasets from six tissues in GTEX, and applied five data correction methods and compared the outputs of these together with the uncorrected dataset. They evaluated the impact of confounding correction methods by calculating different network topological properties such as node density, clustering coefficient and module properties. Additionally, they tested to see  the extent to which the correlated gene pairs from the different methods capture previously published co-expression networks as well as regulatory networks. Overall this is an important effort because of wide use of co-expression analysis, however, the scope of the study is limited as only one data source was considered and the metrics used to evaluate the results rely solely on computationally derived networks. The paper also did not provide a clear recommendation of how one must deal with confounding factors and if at all.

1. Several results are not interpreted. Eg what do we conclude from the observation that CONFETTI and PEER have the largest proportion of null gene-gene correlations. Also the authors use a cutoff of 0.5, but the results could change depending upon the cutoff.

2. Although the GIANT networks were inferred using a systematic machine learning approach, they are still predictions and I am not convinced they alone are good resources to assess the quality of the inferred networks. It would be helpful to use more experimentally derived interactions, e.g. from knock down experiments and chip-seq/chip-chip experiments. Perhaps asking this question for specific cell lines with larger collections of experimentally derived interactions would be better suited? The authors also have another network from Marbach et al, but they seem to use it only for module analysis. Another direction might be to consider simulated datasets where different situations can be better controlled.

3. The overall conclusion that no correction is almost as good as RUVCorr is interesting and worth verifying how broadly this is true. I think considering more datasets and experimentally derived gold standards to show if this is indeed the case would be very beneficial. Perhaps considering model organisms where is more data would be worthwhile.

4. It seems like PEER and CONFETTI are learning sparse networks because of this their performance is low. Is it possible to control for the network size across methods and compute metrics like early precision or F1 score to see if this makes a difference?

5. WGCNA is a widely used method but module identification is a hard problem and results can vary depending upon the method. It might be good to try another method, e.g. a spectral clustering method that was shown to be superior in this study https://www.nature.com/articles/s41592-019-0509-5.

6. The authors attempted to explain the poor performance of CONFETI, by saying that CONFETI is trying to find common genetic variation. Common genetic variation makes sense for eQTL like datasets, but it was not clear how the CONFETI method was capturing this without additional information. The additional comparison that they did to show that CONFETI was capturing different modules seems circular and did not provide additional insight into why CONFETI had lower performance.

7. It might be beneficial to also examine why PEER did not perform well either on these metrics.

8. It seems the authors finding that PC correction is not useful contradicts what was done by Parsana et al. A more in-depth discussion and some experiments to use similar metrics as in Parsana et al would be beneficial and will also show how this study improves on what was done by Parsana et al.

===

Please consider the following questions:

- Are the methods appropriate to the aims of the study, are they well described, and are necessary controls included? If not, please specify what is required.

Additional evaluation metrics, datasets and experimentally derived networks are needed.

- Are the conclusions adequately supported by the data shown? If not, please explain

Yes

- Are sufficient details provided to allow replication and comparison with related analyses that may have been performed? If not, please specify what is required.

Yes

- Does the work represent a significant advance over previously published studies?

No

- Is the paper of broad interest to others in the field, or of outstanding interest to a broad audience of biologists?

This is of broad interest

**Authors Response**

We thank reviewers 1 and 2 for their thoughtful responses and suggestions for our manuscript. We have address all comments, as shown below in our point-by-point response.

Lines number below refer to the revised manuscript without track changes included.

**Reviewer 1 Comments**

This manuscript compares the performances of several confound adjustment methods for co-expression analysis through the applications to six bulk tissue RNA-seq datasets. Some recommendations are provided for the use of these confound adjustment methods, which can be helpful for biologists when conducting co-expression analysis. The manuscript is well written. My major concern is that no significance results are provided to support their comparison results. To name a few:

**Comment 1:** In Fig. 1A and Fig. S2, the densities of Pearson correlation coefficients of various methods are compared, but no p-values are provided. The correlation coefficient difference between any two methods could be simply due to randomness, so I suggest that the authors provide pairwise comparison test results to support their findings.
**Comment 2**: In Fig. 1C and Fig. 1D, are the module sizes and module densities significant different between various methods?
**Comment 3:** In Fig. 2B, the aucodds scores are provided, again, are the differences significant?

We thank Reviewer 1 for their comments. Formal statistical tests have been provided for Fig. 1A, 1B, 1C, and Fig. S2. It is not common practice to conduct pairwise tests for performance evaluation measures^1–3^, so we are reluctant to include these statistics in the manuscript. We understand this is an editorial choice and have provided pairwise tests for performance measures at the end of this document.

For some measures like the AUROC or AUPR, results exceed the 0.50 score we expect for a random classifier. Whether or not differences between adjustment methods are biologically meaningful will be up to the reader, but the consistency of our results across tissue datasets and across evaluation measures suggests to us that the differences we see between adjustment methods are not due to randomness.

**Reviewer 2 Comments**
In this paper, the authors present an interesting and useful analysis of how one must correct bulk RNA-seq expression datasets to remove known and unknown confounding factors before performing co-expression network analysis. To this end the authors obtained datasets from six tissues in GTEX, and applied five data correction methods and compared the outputs of these together with the uncorrected dataset. They evaluated the impact of confounding correction methods by calculating different network topological properties such as node density, clustering coefficient and module properties. Additionally, they tested to see the extent to which the correlated gene pairs from the different methods capture previously published co-expression networks as well as regulatory networks. Overall this is an important effort because of wide use of co-expression analysis, however, the scope of the study is limited as only one data source was considered and the metrics used to evaluate the results rely solely on computationally derived networks. The paper also did not provide a clear recommendation of how one must deal with confounding factors and if at all.

**Comment 1:** Several results are not interpreted. Eg what do we conclude from the observation that CONFETTI and PEER have the largest proportion of null gene-gene correlations. Also the authors use a cutoff of 0.5, but the results could change depending upon the cutoff.

We apologise for the omission, and agree with the reviewer that more description is warranted. We have expanded our explanation on lines 78-81 and lines 93-96.

Performance measures that are affected by choice of global network cutoff are the F-score and the proportion of edges involving TF-target interactions from the DoRothEA database^4^ (the latter is a new analysis included to address Reviewer 2 Comment 2). We varied global network cutoff and recalculated these statistics, and find that our results are robust to choice of cutoff for a gene-gene relationship (Additional File 1: Fig. S10 and Fig. S14).

**Comment 2:** Although the GIANT networks were inferred using a systematic machine learning approach, they are still predictions and I am not convinced they alone are good resources to assess the quality of the inferred networks. It would be helpful to use more experimentally derived interactions, e.g. from knock down experiments and chip-seq/chip-chip experiments. Perhaps asking this question for specific cell lines with larger collections of experimentally derived interactions would be better suited? The authors also have another network from Marbach et al, but they seem to use it only for module analysis. Another direction might be to consider simulated datasets where different situations can be better controlled.

**Comment 3:** The overall conclusion that no correction is almost as good as RUVCorr is interesting and worth verifying how broadly this is true. I think considering more datasets and experimentally derived gold standards to show if this is indeed the case would be very beneficial. Perhaps considering model organisms where is more data would be worthwhile.

We thank Reviewer 2 for their comment and agree that this study would benefit from comparison to a reference dataset that is not a computationally-derived gene network. To address Comments 2 and 3, we have included another analysis comparing our co-expression networks to an external resource of ChIP-seq binding data, TF binding motif analysis, and literature curated gene-gene relationships. We calculated the proportion of total edges in our global co-expression networks that reflect TF-target gene interactions from this resource. Despite using a tissue-agnostic resource, we again find that consistent with our previous analyses known covariate, unadjusted, and RUVCorr-adjusted datasets demonstrate the highest scores, and PEER-adjusted data the lowest. Unlike previous analyses, CONFETI shows intermediate performance on this measure. These results are provided in Figure 2B and text lines 151-160.

To broaden the scope of our analysis beyond GTEx Consortium resources, we also added the prefrontal cortex post-mortem gene expression dataset from the CommonMind Consortium as an additional data source in this study. Although our gold standard references are less well-represented in CMC co-expression networks, differences in performance between adjustment methods are consistent with our results for GTEx tissue datasets.

We have avoided including simulations in the present study because it would require us to make assumptions regarding confounding effects on expression and true biological co-expression networks that are likely to bias our results. Each adjustment method is likely to perform well in simulation scenarios where the assumptions of the approach are met: for example, PC correction requires the underlying biological network to be scale-free, RUVCorr assumes a linear model of biological signal plus systematic and random noise, etc. In the absence of universally-accepted standards regarding the structure of true biological networks or confounding effects, we believe evaluations on real data from orthogonal resources will yield more robust conclusions.

**Comment 4:** It seems like PEER and CONFETTI are learning sparse networks because of this their performance is low. Is it possible to control for the network size across methods and compute metrics like early precision or F1 score to see if this makes a difference?

Thank you for the helpful suggestion! As suggested, we controlled for network size across methods by selecting the top five million gene-gene connections (as measured by absolute Pearson correlation coefficient) from each global co-expression network. We then computed the F-score through comparison of these gene pairs to the GIANT network reference (Additional File 1: Figure S9). Results of this analysis are consistent with other performance measures, although differences between adjustment methods are less pronounced.

**Comment 5:** WGCNA is a widely used method but module identification is a hard problem and results can vary depending upon the method. It might be good to try another method, e.g. a spectral clustering method that was shown to be superior in this study https://urldefense.proofpoint.com/v2/url?u=https-3A__www.nature.com_articles_s41592-2D019-2D0509-2D5&d=DwIGaQ&c=shNJtf5dKgNcPZ6Yh64b-A&r=i7J566dbrPuam2ZqesYXM-fWxEbkDi7X8pdpTT2AR3w&m=6-jVvENRtNx69dh6n1P74KnW2yRBHD2qadnxkhCEyxo&s=nYD9_OryfpzJsN6CIUXkIl1VFKbNRUZpj5hegti2F_E&e= .

We agree with the reviewer that co-expression modules can vary substantially with choice of module detection method. To address this, we have added evaluations of co-expression modules uncovered using independent component analysis (ICA) and multiscale embedded gene co-expression network analysis (MEGENA). ICA is the best performing co-expression module detection method in a recent comprehensive review^3^. MEGENA, developed by a co-creator of WGCNA, is a popular alternative clustering method^5^.

We find that despite notable differences in the properties of modules uncovered using each method (Figure 1), our performance evaluation of co-expression modules is consistent across methods. When aucodds score rankings are combined across the three module detection methods, we again find that CONFETI and PEER-adjusted data score worse on this measure, as compared to other forms of confound adjustment (Figure 2C).

**Comment 6:** The authors attempted to explain the poor performance of CONFETI, by saying that CONFETI is trying to find common genetic variation. Common genetic variation makes sense for eQTL like datasets, but it was not clear how the CONFETI method was capturing this without additional information. The additional comparison that they did to show that CONFETI was capturing different modules seems circular and did not provide additional insight into why CONFETI had lower performance.

The CONFETI method was designed to retain patterns of co-expression associated with genotype, and remove non-genetic sources of variation, by identifying single nucleotide polymorphisms associated with co-expression (i.e. co-expression eQTLs). We provided both genetic and gene expression data in order to apply the CONFETI method. We have included information about this in our updated manuscript on lines 174-180.

All of our evaluation measures will favor co-expression networks that show stronger overall representation of each reference resource. If there are true “co-expression eQTLs” in a dataset, and they are effectively captured by the CONFETI method, it could result in poor overall performance of CONFETI in this study. To understand if modules from CONFETI-adjusted data represent biological co-expression, we tested for evidence of modules in the Marbach et al. (2016) resource of shared targets of a transcription factor (gene groups we would expect to possibly show co-expression relationships).

To more thoroughly evaluate modules uncovered from CONFETI we have added results from hypergeometric tests of gene set enrichment from the Gene Ontology, KEGG, and Reactome pathway databases (Figure S13). Scores are similar to our results from comparison to the Marbach et al. (2016) resource. We do not see strong evidence for CONFETI-derived modules in the Marbach resource or major pathway databases. Results are reported in lines 138-145.

**Comment 7:** It might be beneficial to also examine why PEER did not perform well either on these metrics.

We test PEER adjustment in this study due to its popularity as a hidden confound adjustment method, but it was designed and tested primarily to improve sensitivity in differential expression and cis-eQTL analyses. Our results indicate that PEER is effective in capturing patterns of correlated expression (including biological co-expression) in large expression datasets, and adjusting for PEER factors is not appropriate before co-expression analysis. We have included further explanation to this effect in text lines 167-173.

**Comment 8:** It seems the authors finding that PC correction is not useful contradicts what was done by Parsana et al. A more in-depth discussion and some experiments to use similar metrics as in Parsana et al would be beneficial and will also show how this study improves on what was done by Parsana et al.

We thank Reviewer 2 for their comment and agree that further comparison of our findings to Parsana et al. was needed. To evaluate their co-expression networks, Parsana et al. calculated the false discovery rate of networks constructed using WGCNA and graphical lasso, and find that PC adjustment reduced the FDR. We added an analysis mirroring the structure of Parsana et al., where we have calculated the false discovery rate (and additionally the false negative rate) of WGCNA modules. Despite differences in choice of “ground truth” reference network, we also observe a reduced FDR for modules from PC-corrected data for two out of three shared tissues. We note however that PC correction also results in an inflated FNR, highlighting the need for performance measures that consider both true positive and negative edges in each co-expression network, such as the AUROC or AUPR.

These results are provided as Figure S15 and main text lines 181-192.

**Additional Changes**

Additional changes to the manuscript are as follows:

1. The number of nodes and edges in each global co-expression network have been provided as Additional File 7.
2. We’ve repeated our AUC calculation (Figure 1A) using a balanced true positive/negative reference class of 5,000 gene pairs from each GIANT tissue network, to ensure no effect of class imbalance on our results. Results are largely unchanged.
3. The Marbach et al. (2016) regulatory networks are available through Synapse at <https://www.synapse.org/#!Synapse:syn4974692>, therefore we’ve removed them as Additional Files 5-10 and instead provided this link in the “Availability of data and materials” section.
4. A heatmap to illustrate the overall performance of each adjustment method has been provided as Figure 3.

**References**

1.	Marbach, D. *et al.* Tissue-specific regulatory circuits reveal variable modular perturbations across complex diseases. *Nat Methods* **13**, 366–370 (2016).

2.	The DREAM Module Identification Challenge Consortium *et al.* Assessment of network module identification across complex diseases. *Nat Methods* **16**, 843–852 (2019).

3.	Saelens, W., Cannoodt, R. & Saeys, Y. A comprehensive evaluation of module detection methods for gene expression data. *Nat Commun* **9**, 1090 (2018).

4.	Garcia-Alonso, L., Holland, C. H., Ibrahim, M. M., Turei, D. & Saez-Rodriguez, J. Benchmark and integration of resources for the estimation of human transcription factor activities. *Genome Res.* **29**, 1363–1375 (2019).

5.	Song, W.-M. & Zhang, B. Multiscale Embedded Gene Co-expression Network Analysis. *PLoS Comput Biol* **11**, e1004574 (2015).

**Address to Reviewer 1 Comments: Pairwise Comparisons**

Fig 1D. Intramodular density

BH-adjusted p-values for pairwise Wilcoxon rank sum tests

| **MEGENA** | CONFETI | Known Covariates | None | PC | PEER |
| --- | --- | --- | --- | --- | --- |
| Known Covariates | <2e-16 | - | - | - | - |
| None | <2e-16 | <2e-16 | - | - | - |
| PC | <2e-16 | <2e-16 | <2e-16 | - | - |
| PEER | 6.4e-08 | <2e-16 | <2e-16 | <2e-16 | - |
| RUVCorr | <2e-16 | <2e-16 | <2e-16 | <2e-16 | <2e-16 |

| **WGCNA** | CONFETI | Known Covariates | None | PC | PEER |
| --- | --- | --- | --- | --- | --- |
| Known Covariates | 1.6e-11 | - | - | - | - |
| None | 6.4e-08 | 0.75 | - | - | - |
| PC | <2e-16 | <2e-16 | 1.3e-11 | - | - |
| PEER | <2e-16 | <2e-16 | <2e-16 | <2e-16 | - |
| RUVCorr | <2e-16 | 1.7e-08 | 5.6e-05 | 2.8e-05 | <2e-16 |

Fig 2A. AUROC score

Kruskal-Wallis rank sum test: AUROC ~ Adjustment Method

Chi-squared = 23.951, df=5, p-value=0.0002319

BH-adjusted p-values for pairwise Wilcoxon rank sum tests

|  | CONFETI | Known Covariates | None | PC | PEER |
| --- | --- | --- | --- | --- | --- |
| Known Covariates | 0.0153 | - | - | - | - |
| None | 0.0277 | 0.8048 | - | - | - |
| PC | 0.1327 | 0.0678 | 0.0678 | - | - |
| PEER | 0.3970 | 0.0044 | 0.0323 | 0.0277 | - |
| RUVCorr | 0.0153 | 0.8048 | 0.8048 | 0.0795 | 0.0044 |

Fig 2B. Proportion of edges involving TF-target interactions from DoRothEA database

One-Way ANOVA: Proportion ~ Adjustment Method

F value = 23.951, df=5, p-value= 4.54e-07

P-values for Tukey’s HSD tests

|  | CONFETI | Known Covariates | None | PC | PEER |
| --- | --- | --- | --- | --- | --- |
| Known Covariates | 0.5728894 | - | - | - | - |
| None | 0.9915510 | 0.8915310 | - | - | - |
| PC | 0.8886354 | 0.0953343 | 0.5681055 | - | - |
| PEER | 0.1056715 | 0.0015111 | 0.0273619 | 0.6033568 | - |
| RUVCorr | 0.5184183 | 0.9999993 | 0.8558624 | 0.0789061 | 0.0011760 |

Fig S9. AUPR and F-score for performance evaluation of global co-expression networks

Kruskal-Wallis rank sum test: AUPR ~ Adjustment Method

Chi-squared = 20.552, df=5, p-value= 0.0009841

BH-adjusted p-values for pairwise Wilcoxon rank sum tests

|  | CONFETI | Known Covariates | None | PC | PEER |
| --- | --- | --- | --- | --- | --- |
| Known Covariates | 0.0204 | - | - | - | - |
| None | 0.0375 | 1.00 | - | - | - |
| PC | 0.1366 | 0.1622 | 0.2845 | - | - |
| PEER | 0.3242 | 0.0087 | 0.0375 | 0.0375 | - |
| RUVCorr | 0.0262 | 1.00 | 1.00 | 0.1923 | 0.0204 |

Kruskal-Wallis rank sum test: F-score ~ Adjustment Method

Chi-squared = 26.086, df=5, p-value= 8.586e-05

BH-adjusted p-values for pairwise Wilcoxon rank sum tests

|  | CONFETI | Known Covariates | None | PC | PEER |
| --- | --- | --- | --- | --- | --- |
| Known Covariates | 0.0758 | - | - | - | - |
| None | 0.0758 | 0.1216 | - | - | - |
| PC | 0.1927 | 0.0087 | 0.0087 | - | - |
| PEER | 0.6171 | 0.0237 | 0.0237 | 0.1537 | - |
| RUVCorr | 0.0758 | 0.0710 | 0.0237 | 0.0233 | 0.0237 |

One-Way ANOVA: F-score (5 million edges) ~ Adjustment Method

F value = 3.446, df=5, p-value= 0.012

P-values for Tukey’s HSD tests

|  | CONFETI | Known Covariates | None | PC | PEER |
| --- | --- | --- | --- | --- | --- |
| Known Covariates | 0.0545994 | - | - | - | - |
| None | 0.5448634 | 0.7949784 | - | - | - |
| PC | 0.8781081 | 0.4357189 | 0.9908520 | - | - |
| PEER | 1.0000000 | 0.0517746 | 0.5305461 | 0.8686787 | - |
| RUVCorr | 0.0786271 | 0.9999838 | 0.8710516 | 0.5340893 | 0.0747410 |

Fig 2C. Aucodds score ranking

BH-adjusted p-values for pairwise Wilcoxon rank sum tests

| **WGCNA** | CONFETI | Known Covariates | None | PC | PEER |
| --- | --- | --- | --- | --- | --- |
| Known Covariates | 0.0893 | - | - | - | - |
| None | 0.0156 | 0.1534 | - | - | - |
| PC | 0.0034 | 0.1078 | 0.8296 | - | - |
| PEER | 0.8605 | 6.6e-05 | 6.7e-06 | 1.9e-06 | - |
| RUVCorr | 0.0330 | 0.0893 | 0.8605 | 0.8296 | 0.0001 |
| Kruskal-Wallis rank sum test: Aucodds score rank ~ Adjustment Method  Chi-squared = 40.16, df=5, p-value= 1.387e-07 | | | | | |

| **ICA** | CONFETI | Known Covariates | None | PC | PEER |
| --- | --- | --- | --- | --- | --- |
| Known Covariates | 2.8e-12 | - | - | - | - |
| None | 4.3e-07 | 9.4e-05 | - | - | - |
| PC | 6.8e-09 | 9.4e-05 | 0.86266 | - | - |
| PEER | 6.6e-05 | 4.3e-07 | 0.13848 | 0.06353 | - |
| RUVCorr | 7.2e-11 | 0.20388 | 0.03427 | 0.04321 | 0.00028 |
| Kruskal-Wallis rank sum test: Aucodds score rank ~ Adjustment Method  Chi-squared = 90.891, df=5, p-value < 2.2e-16 | | | | | |

| **MEGENA** | CONFETI | Known Covariates | None | PC | PEER |
| --- | --- | --- | --- | --- | --- |
| Known Covariates | 3.9e-08 | - | - | - | - |
| None | 1.2e-06 | 0.91039 | - | - | - |
| PC | 1.3e-08 | 0.08472 | 0.33738 | - | - |
| PEER | 0.00218 | 5.3e-05 | 0.00055 | 4.7e-07 | - |
| RUVCorr | 9.8e-05 | 0.00055 | 0.00595 | 1.2e-06 | 0.30169 |
| Kruskal-Wallis rank sum test: Aucodds score rank ~ Adjustment Method  Chi-squared = 76.398, df=5, p-value = 4.751e-15 | | | | | |

| **Combined** | CONFETI | Known Covariates | None | PC | PEER |
| --- | --- | --- | --- | --- | --- |
| Known Covariates | <2e-16 | - | - | - | - |
| None | 7.3e-14 | 0.15505 | - | - | - |
| PC | <2e-16 | 0.65235 | 0.28566 | - | - |
| PEER | 0.00013 | 4.4e-14 | 8.9e-09 | 7.3e-14 | - |
| RUVCorr | 2.3e-13 | 0.07571 | 0.72698 | 0.15505 | 1.1e-07 |
| Kruskal-Wallis rank sum test: Aucodds score rank ~ Adjustment Method  Chi-squared = 159.53, df=5, p-value = < 2.2e-16 | | | | | |

**Second round of review**

**Reviewer 1**

My comments have been fully addressed and I have no additional comments.

**Reviewer 2**

The authors have addressed all my concerns and have made more clearer recommendations for practitioners which should be useful. This also leaves open several questions, which will be useful for future research directions. I only have one minor comment. Can all numbers be shown on Fig 3 heatmap?


===
This box is for your report. All comments included here will be passed to the authors.
===
Please consider the following questions:
- Are the methods appropriate to the aims of the study, are they well described, and are necessary controls included? If not, please specify what is required.
Yes.
- Are the conclusions adequately supported by the data shown? If not, please explain
Yes.
- Are sufficient details provided to allow replication and comparison with related analyses that may have been performed? If not, please specify what is required.
Yes
- Does the work represent a significant advance over previously published studies?
Yes, some advance.
- Is the paper of broad interest to others in the field, or of outstanding interest to a broad audience of biologists?
Yes.
